# Association between diagnostic delay and prognosis of pulmonary tuberculosis in Shandong, China: a retrospective study

**DOI:** 10.1186/s12890-022-02101-z

**Published:** 2022-08-12

**Authors:** Xue-han Zhu, Ning-ning Tao, Qian-yun Zhang, Wan-mei Song, Qi-qi An, Si-qi Liu, Yi-fan Li, Fei Long, Huai-chen Li

**Affiliations:** 1 Department of Respiratory and Critical Care Medicine, Shandong Provincial Hospital Affiliated to Shandong First Medical University, 324 Jingwuweiqi Rd, Huaiyin District, Jinan, 250021 Shandong People’s Republic of China; 2 Shandong First Medical University & Shandong Academy of Medical Sciences, 6699 Qingdao Rd, Huaiyin District, Jinan, 250117 Shandong People’s Republic of China; 3 Department of Respiratory and Critical Care Medicine, Shandong Provincial Hospital Affiliated to Shandong University, 324 Jingwuweiqi Rd, Huaiyin District, Jinan, 250021 Shandong People’s Republic of China; 4 Cheeloo College of Medicine, Shandong University, 44 Wenhuaxi Rd, Lixia District, Jinan, 250012 Shandong People’s Republic of China; 5 Shandong Key Laboratory of Infectious Respiratory Diseases, Shandong Provincial Hospital Affiliated to Shandong First Medical University, 324 Jingwuweiqi Rd, Huaiyin District, Jinan, 250021 Shandong People’s Republic of China; 6 Department of Pulmonary and Critical Care Medicine, The Third Affiliated Hospital of Shandong First Medical University (Affiliated Hospital of Shandong Academy of Medical Sciences), 38 Wuyingshan Rd, Tianqiao District, Jinan, 250031 Shandong People’s Republic of China; 7 First College of Clinical Medicine, Shandong University of Traditional Chinese Medicine, 16369 Jingshi Rd, Lixia District, Jinan, 250355 Shandong People’s Republic of China

**Keywords:** Diagnostic delay, Pulmonary tuberculosis, Trend, Poor prognosis

## Abstract

**Background:**

Tuberculosis (TB) is one of the main infectious diseases that seriously threatens global health, while diagnostic delay (DD) and treatment dramatically threaten TB control.

**Methods:**

Between 2005 and 2017 in Shandong, China, we enrolled pulmonary tuberculosis (PTB) patients with DD. DD trends were evaluated by Joinpoint regression, and associations between PTB patient characteristics and DD were estimated by univariate and multivariate logistic regression. The influence of DD duration on prognosis and sputum smear results were assessed by Spearman correlation coefficients.

**Results:**

We identified 208,822 PTB cases with a median DD of 33 days (interquartile range (IQR) 18–63). The trend of PTB with DD declined significantly between 2009 and 2017 (annual percent change (APC): − 4.0%, *P* = 0.047, 2009–2013; APC: − 6.6%, *P* = 0.001, 2013–2017). Patients aged > 45 years old (adjusted odds ratio (aOR): 1.223, 95% confidence interval (CI) 1.189–1.257, 46–65 years; aOR: 1.306, 95% CI 1.267–1.346, > 65 years), farmers (aOR: 1.520, 95% CI 1.447–1.596), and those with a previous treatment history (aOR: 1.759, 95% CI 1.699–1.821) were prone to developing long DD (> 30 days, *P* < 0.05). An unfavorable outcome was negatively associated with a short DD (OR: 0.876, 95% CI 0.843–0.910, *P* < 0.001). Sputum smear positive rate and unfavorable outcomes were positively correlated with DD duration (Spearman correlation coefficients (rs) = 1, *P* < 0.001).

**Conclusions:**

The DD situation remains serious; more efficient and comprehensive strategies are urgently required to minimize DD, especially for high-risk patients.

## Introduction

Tuberculosis (TB) remains one of the leading causes of death and accounted for the greatest number of deaths from a single infectious agent (ranking above human immunodeficiency virus/acquired immunodeficiency syndrome) before the coronavirus (COVID-19) pandemic. Globally in 2020, up to 9.9 million people developed TB and 1.51 million patients died. With 8.5% of global TB patient numbers, China bears the second heaviest TB burden, behind India [1] .

From global efforts (e.g. establishing and implementing Millennium Development Goals [2]), the incidence of TB has decreased steadily since 2000 [1]. Nationally, the incidence of pulmonary tuberculosis (PTB) declined by 28.5% from 72.95/100000 in 2005 to 52.18/100000 in 2016 [3]. Moreover, in China, TB incidence and mortality rates decreased by 3.2% and 7.7%, respectively, on an annual basis from 2005 to 2016, which was a more pronounced decline than worldwide data (2.0% and 3.0%, respectively) [4, 5]. TB is a preventable and treatable infectious disease; up to 85% of TB patients could be treated successfully with a 6-month anti-TB treatment regimen [2]. However, only 47% patients with TB-related symptoms seek medical help in a timely fashion [6], and critically, up to 42% of PTB patients do not seek any medical attention until TB-related symptoms have continued for 1 month or longer [7] .

Diagnostic delay (DD) is a major challenge to PTB control measures, especially in high-burden countries. Delays in diagnosis and PTB treatment result in poor prognosis and high disease transmission [8, 9]. The World Health Organization (WHO) recommended a TB screening program once a patient presented with a cough for ≥ 2 weeks [10]. However, China had DD of 32 days, which obviously did not get the requirement of WHO in term of diagnosis in time [3]. To achieve the WHO targets, an End TB Strategy (a 90% reduction in the TB incidence and a 95% reduction in TB mortality by 2030 when compared with 2015), prompt diagnosis and treatment seems essential in China [2].

Several studies reported that DD in PTB patients was common in China, and caused low decline of PTB incidence [3, 6, 11–15]. In addition, the COVID-19 pandemic partly disrupted TB services, resulting in an increase in DD [16]. Patient factors (e.g. medical care conduct) and medical factors (e.g. medical equipment and human resources) were both proved to be associated with DD [15, 17]. But the leading role was unknown. The specific influence of DD on PTB prognosis is also unclear. To address this, we estimated trends of PTB with DD; we identified an association between patient characteristics and long DD and a relationship between DD and prognosis in PTB patients.

## Methods

### Data inclusion

**Fig. 1:**
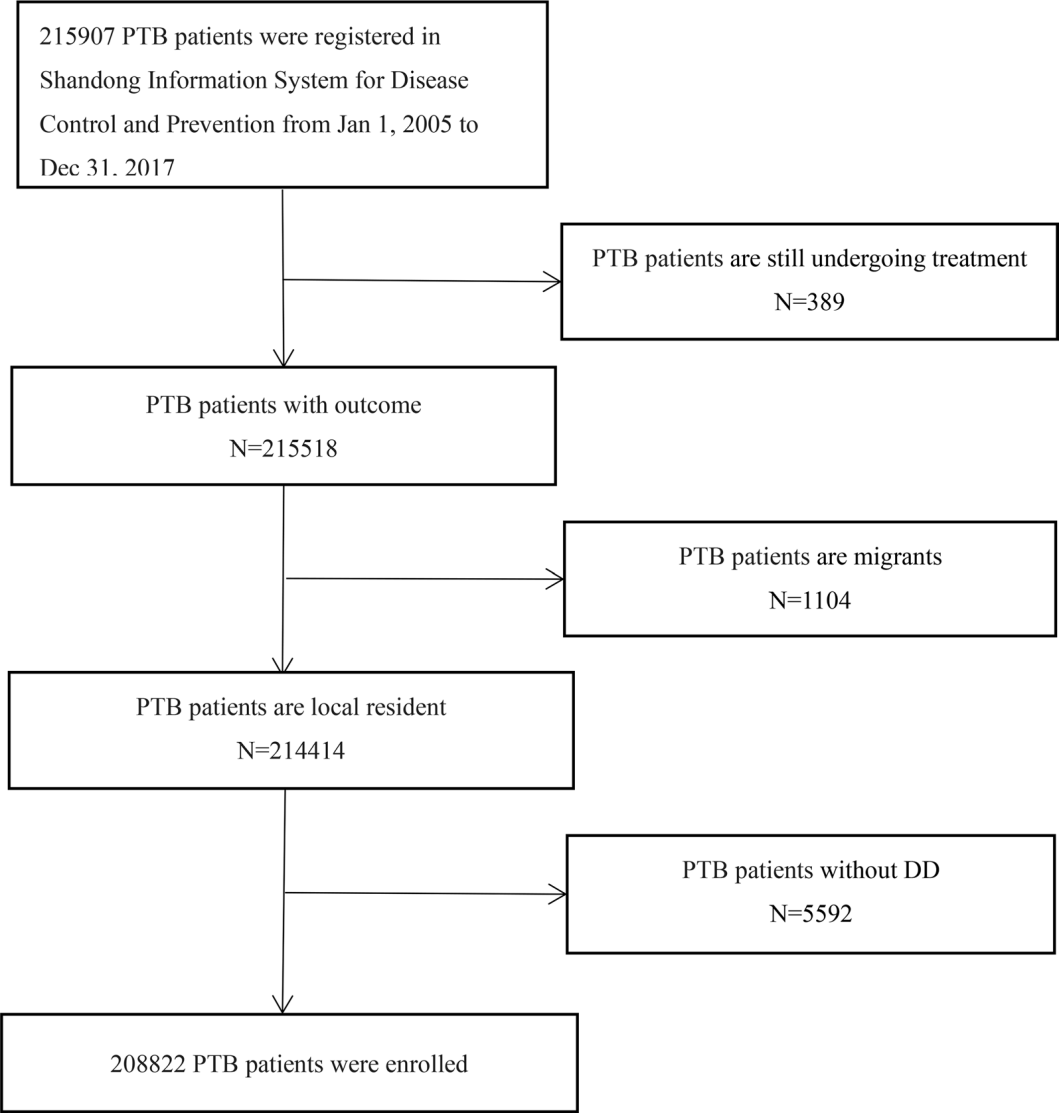
Flow chart of patient inclusion. Legend: PTB = pulmonary tuberculosis; DD = diagnostic delay

Since 2005, the Shandong Information System for Disease Control and Prevention has registered demographic, diagnosis, treatment, and outcome data for TB patients in Shandong Province. Based on convenience and reflecting TB burdens and medical levels, we collected all PTB cases in six cities (Jinan, Linyi, Liaocheng, Yantai, Dezhou, and Jining) in Shandong province from January 2005 to December 2017. Demographic information (sex, age, and occupation), symptom onset date, first medical consultation date, anti-TB treatment date, and clinical characteristics (sputum smear results, previous TB treatment history, and prognosis) were extracted from the database. For some PTB patients, no TB symptoms were accurately recorded until anti-TB treatment initiation, and we hypothesized they were without DD. Also, PTB patients still on treatment at the end of the study period, non-residents, or those without DD were excluded (Fig. 1).

### Case definitions

The following case definitions were used: PTB is defined as any bacteriologically confirmed or clinically diagnosed case of TB involving the lung parenchyma or the tracheobronchial tree. Patients with both pulmonary and extrapulmonary TB should be classified as a case of PTB. New patients: patients never treated for TB or had taken anti-TB drugs for less than 1 month. Previously treated patients: patients who had received anti-TB drugs for at least 1 month in the past.

Favorable outcomes included “cured” and “treatment completed”. “Cured” was defined as a patient who had completed treatment and had at least one bacteriological negative result 1 month before treatment completion. “Treatment completed” referred to patients who had completed treatment, but with no bacteriological results 1 month before treatment completion.

Unfavorable outcomes included those who had “died”, had “treatment failure” and were “not evaluated”. “Died” referred to a patient who had died for whatever reason during the study period. “Treatment failure” referred to anyone whose bacteriological results remained positive after 5 months of treatment. “Not evaluated” included cases who were lost to follow-up and whose treatment outcomes were unknown during the study [18–20] .

PTB patients enrolled in study were generally identified passively when patients self-present with symptoms. Therefore, we defined DD as the time from the first onset of symptoms to the initiation of anti-TB treatment [3, 21, 22]. It comprised two phases: patient delay (time from the first onset of symptoms to the first medical consultation) and provider delay (period from the first medical consultation to anti-TB treatment initiation). For analysis, DD was categorized as short (≤ 30 days) and long (> 30 days) [3, 23].

### Statistical analysis

Continuous variables were represented by IQR (interquartile range) and categorical variables was expressed using by percentages. Univariate and multivariate logistic regression models were used to establish associations between clinical and demographic characteristics and long DD, and were displayed as crude and adjusted odds ratios (OR and aOR, respectively) and 95% confidence intervals (CI).

Correlations were assessed by Spearman rank correlation analysis. Spearman correlation coefficients (rs) ranged from − 1 to + 1. A rs < 0 or > 0 revealed positive or negative relations, respectively, while rs = 0 indicated no significant correlation between two variables [24].

The Joinpoint regression model was used to analyze temporal trends of PTB with DD. Annual percent changes (APCs) represented changes within a specific period. An “increasing or decreasing” trend was defined if APC significantly different from zero (APC > 0 = an increasing trend and APC < 0 = a decreasing trend). Otherwise, it was defined as a “stable” trend [25, 26].

Data were analyzed and managed in SPSS software (version 26.0) and Joinpoint software (version 4.9.0). Significance was accepted at *p* < 0.05.

### Ethics approval and consent to participate

Before data analysis and reporting, all TB patient personal identifiers were removed, therefore informed consent was not required. An informed consent waiver and ethical approval were obtained from the Ethics Committee of Shandong Provincial Hospital Affiliated to Shandong First Medical University, China. The study was conducted in accordance with the Declaration of Helsinki.

## Results

Overall, 215907 PTB patients were recorded in the previous 13 years in Shandong Province. In total, 7085 PTB patients did not meet inclusion criteria for the following reasons: still on treatment by the end of study period (n = 389), non-residents (n = 1104), and without DD (n = 5592). Therefore, 208822 PTB patients with DD were finally selected.

### PTB patients with DD-trends

**Fig. 2:**
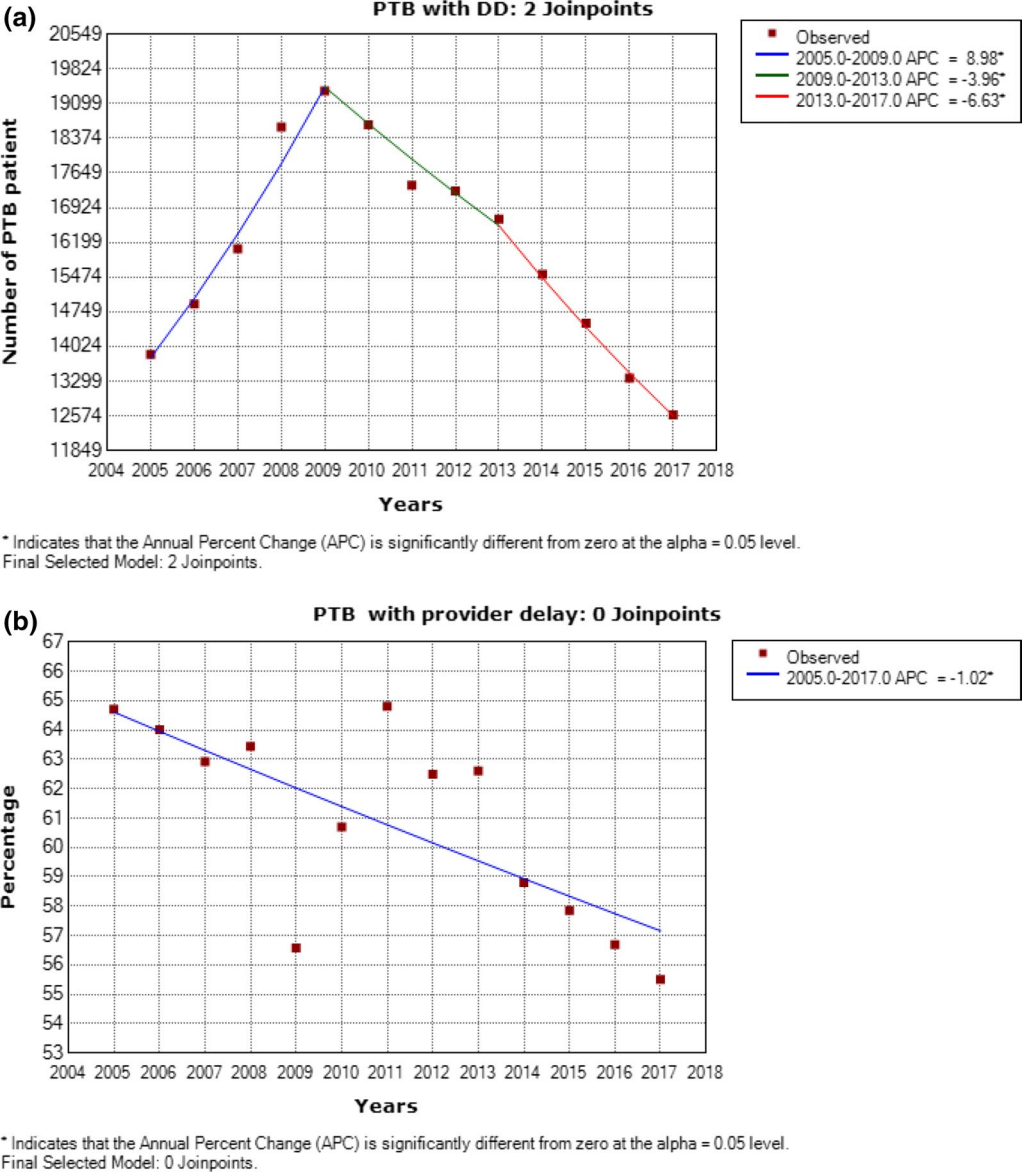

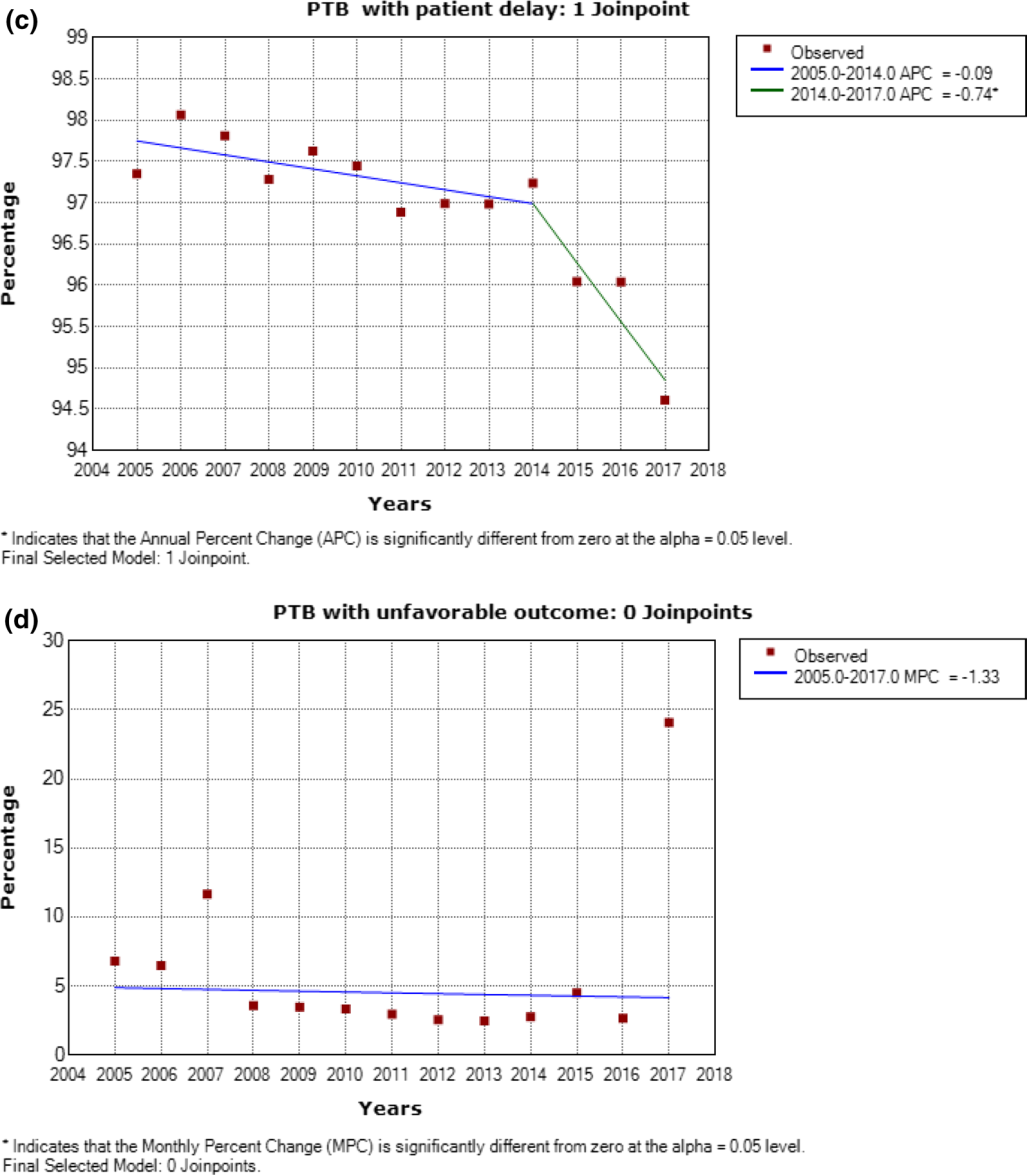
Annual percentage changes of PTB patients with DD in Shandong, China, 2005–2017.** a**) PTB with DD;** b** PTB with provider delay;** c** PTB with patient delay;** d** PTB with unfavorable outcome. Legend: PTB = Pulmonary tuberculosis; DD = Diagnostic delay; Unfavorable outcome is defined as “died”, “treatment failed” and “not evaluated”. *Indicate that the Annual Percent Change (APC) is significantly different from zero at the alpha = 0.05 level or the AAPC is significantly different from zero at the alpha = 0.05 level

In patients, the highest prevalence rate of PTB with DD was observed in 2009 (n = 19362). A significant decrease of PTB with DD was observed between 2009 and 2017 (APC—4.0%, *P* = 0.047, 2009–2013; APC—6.6%, *P* = 0.001, 2013–2017). From 2005 to 2017, PTB with provider delay decreased significantly (APC—1.0%, *P* = 0.006). However, no significant change in the trend of PTB with patient delay was observed from 2005 to 2014 (APC—0.1%, *P* = 0.095). We also observed far more PTB with patient delay (97.0%) than provider delay (61.1%) among patients. Furthermore, patients with unfavorable outcomes did not change significantly during the study period (APC—1.3%, *P* = 1) (Fig. 2).

### Characteristics of PTB patients with DD

Between 2005 and 2017, 208822 PTB patients had DD, of which 70.2% were male, 56.8% were aged > 45 years, 81.1% were farmers, and 90.0% were newly treated in Shandong Province. The median DD between first symptom onset until anti-TB treatment initiation was 33 days (IQR 18–63). From both univariate and multivariate adjustment analysis, long DD was significantly associated with PTB patients aged > 45 years (OR: 1.407, 95% CI 1.373–1.443, aOR: 1.223, 95% CI 1.189–1.257, *P* < 0.001, aged 46–65; OR: 1.524, 95% CI 1.483–1.566, aOR: 1.306, 95% CI 1.267–1.346, *P* < 0.001, aged > 65), farmers (OR: 1.834, 95% CI 1.755–1.916, aOR: 1.520, 95% CI 1.447–1.596, *P* < 0.001), and previously treated patients (OR: 1.875, 95% CI 1.812–1.941, aOR: 1.759, 95% CI 1.699–1.821, *P* < 0.001) (Table 1).

**Table 1:** Demographic and clinical characteristics of 208,822 PTB patients with DD in Shandong, China, 2005–2017

Characteristics	Total (N = 208,822)	Median (days)	DD ≤ 30 Days (N = 87,896)	DD > 30 Days (N = 120,926)	Univariable		Multivariable	
					*P* value	OR (95% CI)	*P* value	aOR (95% CI)
*Age (years)*								
≤ 25	38,765 (18.5%)	31 (15,59)	18,672 (21.2%)	20,093 (16.6%)	Reference	Reference	Reference	Reference
26–45	51,491 (24.7%)	32 (17,62)	22,937 (26.1%)	28,554 (23.6%)	*P* < 0.001	1.157 (1.127–1.188)	*P* < 0.001	1.046 (1.016–1.076)
46–65	70,570 (33.8%)	33 (20,65)	28,074 (32.0%)	42,496 (35.2%)	*P* < 0.001	1.407 (1.373–1.443)	*P* < 0.001	1.223 (1.189–1.257)
> 65	47,996 (23.0%)	34 (20,67)	18,213 (20.7%)	29,783 (24.6%)	*P* < 0.001	1.524 (1.483–1.566)	*P* < 0.001	1.306 (1.267–1.346)
*Sex*								
Male	146,538 (70.2%)	33 (18,63)	61,479 (69.9%)	85,059 (70.3%)	Reference	Reference		
Female	62,284 (29.8%)	33 (18,64)	26,417 (30.1%)	35,867 (29.7%)	*P* = 0.052	1.019 (1.000–1.038)		
*Occupation*								
Student	8516 (4.1%)	25 (9,49)	4737 (5.4%)	3779 (3.1%)	Reference	Reference	Reference	Reference
Farmer	169,365 (81.1%)	33 (19,64)	68,764 (78.2%)	100,601 (83.2%)	*P* < 0.001	1.834 (1.755–1.916)	*P* < 0.001	1.520 (1.447–1.596)
Worker	10,410 (5.0%)	31 (14,62)	5048 (5.7%)	5362 (4.4%)	*P* < 0.001	1.331 (1.257–1.410)	*P* < 0.001	1.220 (1.149–1.296)
Service stratum	10,003 (4.8%)	32 (13,65)	4689 (5.4%)	5314 (4.4%)	*P* < 0.001	1.421 (1.340–1.505)	*P* < 0.001	1.297 (1.221–1.378)
Other	10,525 (5.0%)	33 (16,65)	4658 (5.3%)	5867 (4.9%)	*P* < 0.001	1.579 (1.491–1.672)	*P* < 0.001	1.334 (1.254–1.419)
*TB treatment history*								
Newly treated	191,896 (91.9%)	32 (17,62)	83,004 (94.4%)	108,892 (90.0%)	Reference	Reference	Reference	Reference
Previously treated	16,926 (8.1%)	61 (28,333)	4892 (5.6%)	12,034 (10.0%)	*P* < 0.001	1.875 (1.812–1.941)	*P* < 0.001	1.759 (1.699–1.821)

*PTB* Pulmonary tuberculosis, *DD* Diagnostic delay, *OR* Crude odds ratio, *aOR* Adjusted odds ratio, *CI* Confidence interval

### DD and smear results

**Fig. 3:**
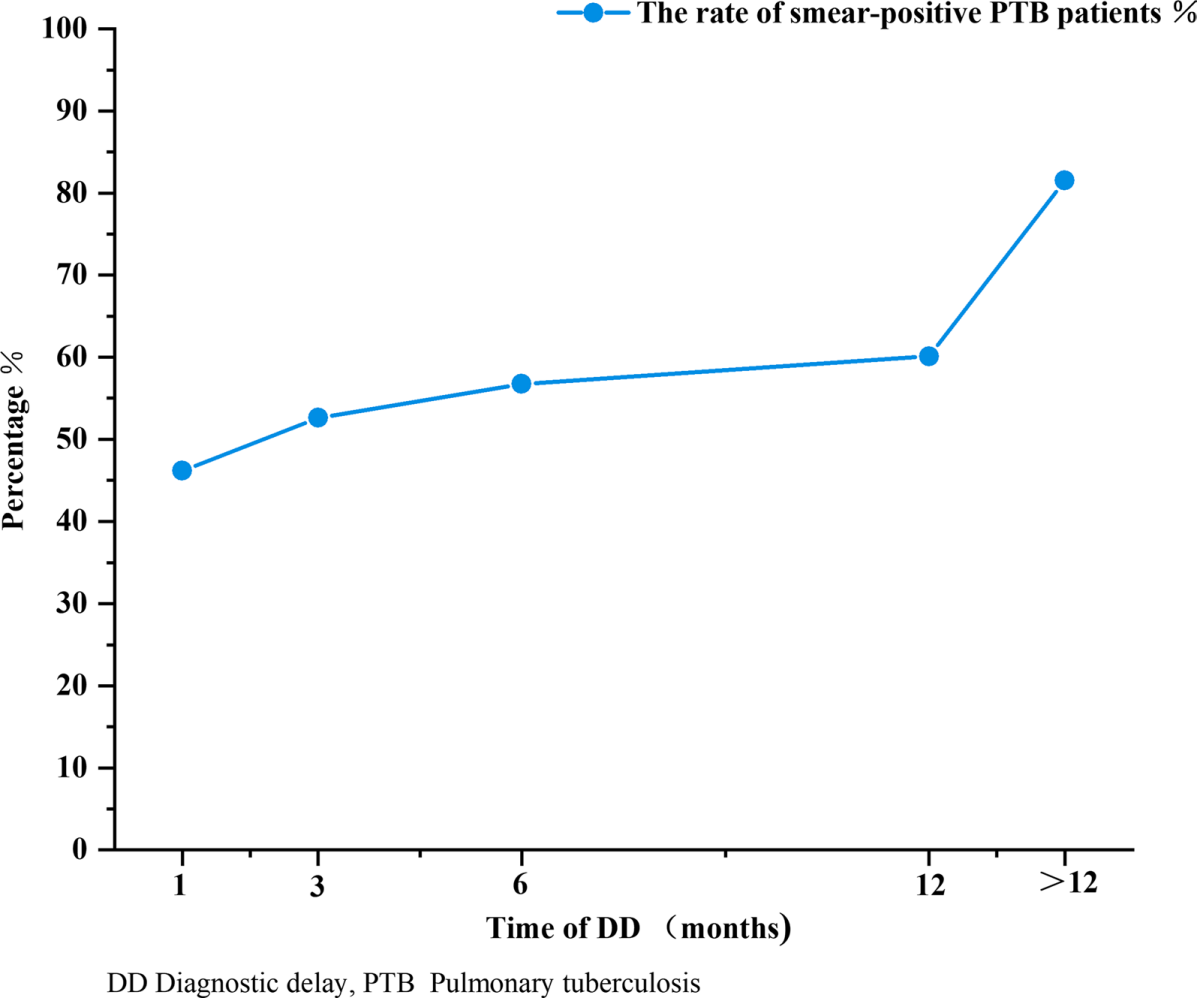
The sputum smear results of PTB patients according to different DD duration. Legend: PTB = Pulmonary tuberculosis; DD = Diagnostic delay

In PTB patients, sputum smear positive rate was positively associated with the DD duration (rs = 1, *P* < 0.001). Among PTB patients with a DD duration exceeding 1 year, up to 81.5% of cases had positive smears (Fig. 3).

### DD and prognosis

Approximately 5.8% of PTB patients with long DD had unfavorable outcomes, which were higher than PTB patients with short DD (5.1%). Unfavorable outcomes were negatively associated with a short DD (OR: 0.876, 95% CI 0.843–0.910, *P* < 0.001). Treatment failure (OR: 1.174, 95% CI 1.047–1.316, *P* = 0.006), non-TB death (OR: 1.614, 95% CI 1.470–1.771, *P* < 0.001), and TB death (OR: 1.587, 95% CI 1.342–1.878, *P* < 0.001) were associated with long DD (Table 2).

**Table 2:** Treatment outcomes of PTB patients with DD ≤ 30 days and DD > 30 days

	No. of patients		*P*	OR (95%CI)
	DD ≤ 30 days (87,896)	DD > 30 days (120,926)		
Unfavorable outcome	4518 (5.1)	7044 (5.8)	*P* < 0.001	0.876 (0.843,0.910)
*Died*				
Non-TB death	655 (0.8)	1425 (1.2)	*P* < 0.001	1.614 (1.470,1.771)
TB death	199 (0.2)	434 (0.4)	*P* < 0.001	1.587 (1.342,1.878)
Treatment failed	478 (0.5)	768 (0.6)	*P* = 0.006	1.174 (1.047,1.316)
Not evaluated	3185 (3.6)	4417 (3.6)	*P* = 0.716	1.009 (0.963,1.057)
Favorable outcome	83,378 (94.9)	113,882 (94.2)	Reference	Reference

*DD* Diagnostic delay, *TB* Tuberculosis, *PTB* Pulmonary tuberculosis

Favorable outcome including “cured” and “treatment completed”

Unfavorable outcome is defined as “died”, “treatment failed” and “not evaluated”

“Died” means a patient died for whatever reason during the study period. It combined with TB death and non-TB death. Non-TB death means that TB was not the main cause of death. “Treatment failed” means anyone whose bacteriological result remains positive after 5 months treatment. “Not evaluated” includes cases who are lost to follow-up and whose treatment outcome is unknown during the study period

**Fig. 4:**
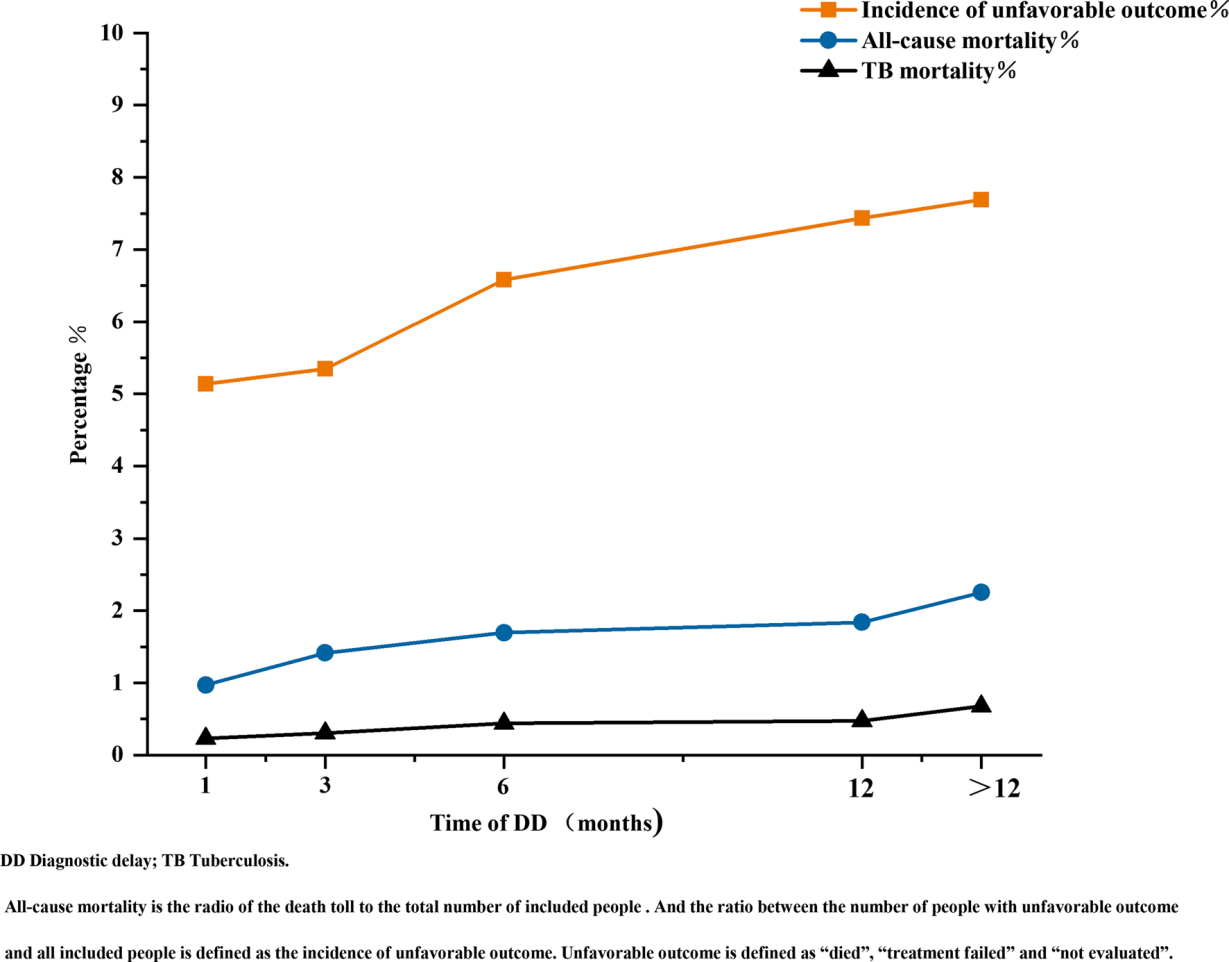
The outcome of PTB patients according to different DD duration. Legend: TB = Tuberculosis; DD = Diagnostic delay. All-cause mortality is the radio of the death toll to the total number of included people. And the ratio between the number of people with unfavorable outcome and all included people is defined as the incidence of unfavorable outcome. Unfavorable outcome is defined as “died”, “treatment failed” and “not evaluated.”

Correlation analyses showed that an unfavorable outcome (rs = 1, *P* < 0.001), all-cause mortality (rs = 1, *P* < 0.001), and TB mortality (rs = 1, *P* < 0.001) were positively associated with the DD duration (Fig. 4).

## Discussion

We provided further information on PTB trends with DD and risk factors related to long DD in Shandong, China. Our main findings showed that an unfavorable prognosis and sputum smear positive rate were positively correlated with DD duration. PTB patients > 45 years old, with a previous treatment history, and farmers were at high risk of developing long DD. DD was more common in PTB patients than that in medical part. Although the PTB incidence has decreased in China, a DD with a median of 33 days cannot be ignored.

We observed a declining trend in PTB with DD. Since the 1990s, the government has introduced several TB control policies, which may have contributed to these declines. With economic support from the World Bank, a program called the Convergence Management System was adopted under the National TB program of the Chinese Ministry of Health. The government provided free TB diagnosis (including sputum tests and chest X-rays) and treatment (first line anti-TB drugs) opportunities for infectious TB patients who were diagnosed and treated in county TB dispensaries (CTD). Doctors who referred suspicious or diagnosed TB patients to CTD received a bonus [6]. Moreover, from 2003, the rural cooperative medical system in China was reconstructed and the Urban Resident Basic Health Insurance scheme established, which increased financial support for TB control. Since 2004, the universal coverage of directly observed treatment and short course chemotherapy (DOTS) strategy improved diagnosis and treatment for TB [6, 27]. Since 2005, PTB patients with negative sputum smear results could receive free treatment. Critically, these strategies have contributed to a declining trend of PTB with DD.

However, patient delay remains an important reason for DD. Although anti-TB drugs are free, other diagnostic and therapeutic methods, including computed tomography and adjuvant therapy are expensive and may prohibit poorer patients from seeking treatment. Also, public awareness of TB is inadequate; erroneous notions of TB still prevail, such as TB stigma [7, 28]. These factors may contribute to poor patient delay.

We observed a positive relationship between sputum smear positive rate and long DD, consistent with previous studies [28, 29]. Long DD may generate a high mycobacterial load and increased *Mycobacterium tuberculosis* (*M.tb*) excretion, leading to high positive smear rates. Additionally, individuals inevitably come into contact with undiagnosed TB patients who may carry *M.tb*, thus increasing TB transmission [11, 30].

Unfavorable outcomes were related to long DD and positively associated with the DD duration. Previous evidence indicated that for the greatest delays, patient outcomes were worse. DD in PTB may cause more severe damage to the lungs [31], therefore such patients are at a high risk of respiratory complications, such as bronchiectasis and hemoptysis, which may complicate anti-TB treatments and impede recovery [32–34]. Moreover, previous evidence also reported that DD was a major risk factor for TB-related death [35]. Therefore, PTB patients can benefit from a prompt diagnosis and treatment.

Patients aged > 45 years old, farmers, and those with a previous TB treatment history were more likely to suffer long DD. Patients > 45 years old accounted for a large percentage of PTB cases with DD. Middle-aged adults, who support children and aging parents, have suffered with economic and social stress which exacerbated their own long-neglected health conditions. In addition, the majority of PTB patients > 65 years old always had atypical symptoms or several complications, which perplexed medical providers and resulted in DD [30]. Previous research also indicated that aging populations were problematic for TB control [36, 37]. Farmers were also likely to suffer with long DD, which may be associated with low education levels and a less developed socioeconomic infrastructure [6, 7, 38]. TB is a disease of poverty [5], therefore less developed socioeconomic infrastructures invariably mean lower purchasing power for medical services and a lack of medical facilities. A previous study reported that long DD was related to low education levels; the lack of a good education may lead to insufficient public health and TB-associated knowledge. Moreover, it was previously reported that dwelling areas was an important determinant of patients in seeking and receiving care for TB [39]. Similarly in a previous study, a TB treatment history was a risk factor for DD [24]. TB can result in chronic lung injury, with anti-TB treatments confirmed as a high-risk factor for pulmonary function deterioration [40]. Patients with a TB treatment history were more likely to suffer other respiratory disorders, which may have been confused with a TB diagnosis [11, 38].

Globally in 2020, 56% of TB patients were adult men [1]. This cohort appeared to bear the heaviest economic burden; interestingly, 70% of patients in our study were men. However, we observed no significant associations between sex and DD, in contrast with previous studies [6, 41].

Our study had some limitations. Firstly, we only studied PTB patients in an eastern coastal province of China, populations across the Midwest were not included. Secondly, as a retrospective study, we derived all relevant information from the Information System for Disease Control and Prevention, thus recall or diagnostic suspicion bias were unavoidable. And certain clinical information (e.g. classification of PTB, complications, drug susceptibility testing results) and demographic information (e.g. education, economic status, marital status, absence of caregivers) were not available on all patients. Few information of medical part in DD (e.g. self-medication, place of initial consult) were on the record. Thirdly, we only included PTB patients, therefore our results may not be applicable to those only with extrapulmonary TB, which are needed to further analyze.

## Conclusions

Although PTB patient numbers with DD declined from 2009 to 2017, the DD situation remains serious. Long DD will generate more positive sputum smears rate and poorer prognosis. Therefore, medical workers must be more aware of TB and use high sensitivity molecular tools to minimize provider delay [42]. Also, popularizing TB knowledge is necessary, especially for high-risk patient populations. Furthermore, the government should increase investment for improved health service delivery and increase investment in medical research and development [43]. This way, we can sustain or accelerate the decline in DD and achieve the 2030 targets.

## Data Availability

The datasets generated and/or analysed during the current study are available from the corresponding author on reasonable request.

## Abbreviations

TB: Tuberculosis
DD: Diagnostic delay
PTB: Pulmonary tuberculosis
IQR: Interquartile range
APC: Annual percent change
aOR: Adjusted odds ratio
OR: Odds ratio
CI: Confidence interval
rs: Spearman correlation coefficients
WHO: World Health Organization
CTD: County TB dispensaries
DOTS: Directly observed treatment and short course chemotherapy
*M.tb*: *Mycobacterium tuberculosis*
